# Status and influencing factors of active health behaviors in high-risk stroke populations: a cross-sectional study

**DOI:** 10.3389/frhs.2026.1838501

**Published:** 2026-06-24

**Authors:** Lingmei Ruan, Chengjian Zhang, Rong Du, Shuang Liu, Mengfan He, Li Pu, Lixiang Deng, Hongwei Yang, Yun Bao, Shaoxiang Zhou

**Affiliations:** 1 Nursing College, Medical Department, Dali University, Dali City, Yunnan Province, China; 2 Panzhihua College of Health Care, Panzhihua University, Panzhihua City, Sichuan Province, China; 3 Department of Neurology, Panzhihua Central Hospital, Panzhihua, Sichuan, China; 4 Basic Medical Department of Panzhihua University, Panzhihua City, Sichuan Province, China

**Keywords:** active health behaviors, cross-sectional study, health promotion, high-risk population, primary prevention, stroke

## Abstract

**Background:**

This study aims to investigate active health behaviors among stroke high-risk populations in Panzhihua, Sichuan Province, and to identify the key sociodemographic, behavioral, and environmental factors associated with these behaviors. By characterizing the current status and patterns of active health behavior adoption in this population, the study seeks to inform the development of evidence-based, targeted primary prevention strategies for stroke.

**Methods:**

This study employed a cross-sectional design. Between July and October 2025, a total of 616 stroke high-risk individuals were recruited from community health centers in Panzhihua. Data were collected using a self-administered questionnaire comprising the following instruments: a general information questionnaire, the Chronic Disease Active Health Behavior Scale, the Health Motivation Behavior Scale, the Health Literacy Questionnaire, the Medical Social Support Scale, and the General Self-Efficacy Scale.

**Results:**

Among the 616 participants recruited, all 616 (100%) completed the questionnaire, with 319 males (51.8%) and 297 females (48.2%). For the population at high risk of stroke, the overall mean score of proactive health behaviors was 55.90 ± 7.85, and the mean item score was 2.79 ± 0.39. According to the reference scoring criteria of the scale, a total score ≥ 80 indicates good proactive health behaviors, while a total score < 60 indicates poor proactive health behaviors. The proactive health behavior level of this population was lower than the medium level. Univariate analysis showed that there were statistically significant differences in the scores of proactive health behaviors among participants with different ages, smoking statuses, alcohol consumption levels, residential locations, sources of livelihood, numbers of chronic comorbidities, frequencies of physical examinations, and whether they received training or obtained self-learning knowledge related to stroke (*P* < 0.05). Multiple linear regression analysis demonstrated that health literacy, medical social support, general self-efficacy, age, smoking, source of livelihood, and physical examination status had significant effects on proactive health behaviors among the population at high risk of stroke (*P* < 0.05).

**Conclusions:**

The level of active health behaviors among stroke high-risk populations in the Panzhihua area needs to be improved. As a cross-sectional study, the present study collected data at a single time point and identified the significant correlations between active health behaviors and multiple influencing factors, including health literacy, medical social support, and self-efficacy. However, the samples of this study were recruited only from the Panzhihua area, which may be a problem of limited representativeness and makes it difficult to observe the dynamic changes of individual behaviors over time. Therefore, the conclusions drawn in this study are only applicable to similar regions, and their generalizability may be limited. Future research should carry out multi-center, longitudinal, interventional, and mixed-methods studies, with a focus on translating health literacy into practice, strengthening social support systems, enhancing self-efficacy, and implementing personalized primary prevention management for populations with different characteristics.

## Introduction

Stroke represents a major global public health concern, characterized by high incidence, high disability rate, high mortality rate, high recurrence rate, and heavy economic burden. In 2021, stroke ranked as the third most common cause of death globally, and the fourth leading contributor to disability-adjusted life years (DALYs) (1). Data from the 2021 Report on Stroke Prevention and Control in China (2) indicates that 4.09 million new stroke cases were reported in China, and stroke has become the second leading cause of death among rural residents and the third leading cause of death among urban residents in the country.

The World Health Organization points out that primary prevention is the most effective strategy for reducing stroke incidence, and healthy behaviors can reduce 80% of stroke risk and 50% of stroke incidence (3). To further promote the transformation of China's healthcare development philosophy from “treatment-centered” to “health-centered”, the concept of “active health” has emerged. This concept emphasizes that individuals are the “first responsible persons” for their own health, and advocates the achievement of disease prevention and health management through giving full play to individual initiative and sustaining participation in healthy behaviors (4, 5). As the practical pathway for implementing active health, active health behavior is the key link that translates the conceptual framework into tangible health benefits (6).

Although the importance of active health behaviors in the primary prevention of populations at high risk of stroke has been widely recognized, existing research on the level of active health behaviors and their influencing factors among this population remains limited (7). Investigating the level of active health behaviors in this population is particularly critical, as the identified influencing factors can provide a basis for developing targeted interventions, which in turn improves health behaviors and ameliorates health outcomes. To address this research gap, this study introduces the concept of “active health behaviors” into the primary prevention research of stroke high-risk populations, systematically investigates the actual level of active health behaviors in this population, and conducts an in-depth analysis of its multi-level and multi-dimensional complex influencing factors, so as to identify key intervention targets and implement precise and personalized health management programs, thereby meeting the urgent practical requirement of transforming from passive treatment to proactive health management.

## Objects and methods

### Participants

From July to October 2025, ten townships in Panzhihua were selected. Stratified by township, residents aged 40 years and above were screened by Panzhihua Central Hospital in collaboration with local communities in accordance with the stroke high-risk population screening program. Using the stratified random sampling method (8), ten townships were randomly selected from communities with aggregated high-risk stroke populations in Panzhihua, and at least 20 high-risk stroke individuals were recruited as study participants from each age stratum (40∼49 years, 50∼59 years, 60∼69 years, 70∼79 years, and 80 years and above). Based on the principle and standard of the rough estimation method for sample size (9), the sample size should be 5–10 times the number of variables, and 10 times the number of independent variables was adopted in this study. A total of 42 variables were included in this study, so the required sample size was 420 cases. Considering a 10% rate of invalid questionnaires, the minimum sample size was determined to be 462 cases, and 616 cases were ultimately included in this study.

### Inclusion criteria

Based on the Stroke High-Risk Population Screening Program, the inclusion criteria were as follows: (1) age ≥ 40 years; (2) classified as high-risk for stroke based on the “8 + 2” risk assessment scale promoted by the Stroke Prevention and Treatment Project Committee of the National Health Commission of China (10), defined as having three or more of the eight primary risk factors (e.g., hypertension, dyslipidemia, diabetes, atrial fibrillation, or a history of transient ischemic attack) or a prior history of stroke; (3) clear consciousness and ability to communicate effectively; and (4) provision of informed consent to participate voluntarily in the study.

### Exclusion criteria

The exclusion criteria were as follows: (1) a diagnosis of mental illness or significant cognitive impairment; (2) the presence of other malignant organic diseases; (3) concurrent participation in another clinical study; and (4) unwillingness to provide informed consent for the survey.

### Ethics

This study has obtained ethical review approval from the Scientific Research Ethics Committee of Panzhihua Central Hospital (Approval No. 2025-096). All participants have signed written informed consent, and the study has been conducted in strict compliance with the ethical principles set forth in the Declaration of Helsinki. Prior to signing the informed consent, researchers provided participants with a detailed explanation of the study objectives and procedures, explicitly stated that participation is completely voluntary, and informed participants that they may withdraw from the study at any time during the research process. The medical history and personal information of participants were extracted from the Chinese Website for Cardiovascular and Cerebrovascular Diseases. All collected data were anonymized and kept strictly confidential to ensure that no individual participant can be identified.

### Data collection instruments

A cross-sectional survey was conducted for data collection. The instruments included a socio-demographic questionnaire, the Chronic Disease Active Health Behavior Scale, the Chinese version of the Health Behavior Motivation Scale, the Chinese version of the Health Literacy Questionnaire, the Medical Social Support Scale, and the General Self-Efficacy Scale.

### General information questionnaire

A socio-demographic questionnaire was developed based on a comprehensive literature review and consultations with nursing experts. It collected information on participants' characteristics, including age, gender, ethnicity, education level, marital status, occupation, monthly income, place of residence, living arrangements, source of income, type of health insurance, physical examination history, personal and family history of stroke, smoking and alcohol consumption status, and medication use.

### Proactive health behavior scale for patients with chronic diseases

The Proactive Health Behavior Scale for Patients with Chronic Diseases (PHBS-CD) was developed by Chen Chaoyi et al. (11) in 2024, with a Cronbach's *α* coefficient of 0.904. This scale demonstrates good reliability and validity, and can be used as an assessment tool for medical staff to evaluate the self-managed health behaviors of patients with chronic diseases. The scale comprises five dimensions, namely personal health responsibility, psychological stress coping, interpersonal support, exercise behavior, and nutritional dietary behavior, with a total of 20 items. It adopts the 5-point Likert scoring method, with a total score of 100 points. A total scale score of 80 or higher indicates good proactive health behavior, while a score below 60 indicates poor proactive health behavior. However, since this study was based on cross-sectional survey data of chronic diseases during the scale development phase, it may lead to item comprehension bias. Currently, Wang Mengdi (12) have developed a specific scale for proactive health behavior among populations at high risk of stroke, but this scale lacks a unified standard for classification criteria. Future researchers may further improve the relevant scoring instructions based on the application of the scale.

### The Chinese version of health behavior motivation scal

The Chinese Version of Health Behavior Motivation Scale (HBMS) was developed by Jiang Mei et al. (13) in 2024. This scale comprises 5 dimensions and 30 items in total, whose reliability and validity meet psychometric standards, providing a specialized tool for the scientific and effective assessment of the health behavior motivation level of community-dwelling older adults. The total score of the HBMS ranges from 30 to 150, with higher scores indicating higher levels of health behavior motivation. The test-retest reliability of the Chinese version of the HBMS is 0.882, demonstrating good sensitivity and specificity.

### The Chinese version of the health literacy questionnaire

Developed under the guidance of the conceptual model of health literacy by Sørensen (14), the questionnaire was rigorously translated and evaluated for reliability and validity in 2024 by Chinese scholars Lin Ying et al. (15), resulting in the Chinese version of the Health Literacy Questionnaire (HLS-Q16 Chinese version). The Cronbach's *α* coefficient of the total Chinese version of HLS-EU-Q16 is 0.923. The scale comprises 3 dimensions and 16 items: Items 1–7 belong to the healthcare dimension, Items 8–12 fall into the disease prevention dimension, and Items 13–16 are classified under the health promotion dimension. Each item is answered with one of four options: “very easy”, “easy”, “difficult” and “very difficult”. Among these, “very easy” and “easy” are scored as 1 point, “very difficult” and “difficult” are scored as 0 point, and responses of “don't know/refuse” are coded as missing values. Based on the total score of the scale, health literacy levels are divided into three grades: inadequate health literacy (0∼8 points), limited health literacy (9∼12 points) and sufficient health literacy (13∼16 points).

### The Chinese version of medical outcomes study social support survey

The Medical Outcomes Study Social Support Survey (MOS-SSS) was developed by Sherbourne & Stewart in 1991 (16). In 2004, Doris S. F., Diana T. E. Lee and Jean Woo (The Chinese University of Hong Kong) translated the scale into a traditional Chinese version (17)^,^ the traditional Chinese version of MOS-SSS (C), whose test-retest reliability all exceeds 0.7. This questionnaire consists of 4 dimensions and 20 items. The first item is a subjective question that measures the size of the patient's support network, while items 2–20 are objective items, scored from 1 to 5 according to the frequency of occurrence. After being converted via a standardized formula, the score of each dimension ranges from 0 to 100, with higher scores indicating better social relationships. In 2015, Chen Cuiping (18) conducted a retest of the reliability and validity of the Simplified Chinese version of the scale, and the obtained Cronbach's *α* coefficient was 0.937, indicating that the scale boasts sound reliability and validity.

### General self-efficacy scale

This scale was developed by Bandura (19) in 1977, Shen (20) conducted a retest of reliability and validity in 2004, and the Cronbach's *α* coefficient of internal consistency for the scale was 0.87, and it adopts a 4-point Likert scoring method. The total score of the scale ranges from 10 to 40, with higher scores indicating stronger individual self-efficacy.

Regarding scale permissions, the Medical Social Support Scale and General Self-Efficacy Scale are publicly available for academic use. The remaining three scales (Chronic Disease Active Health Behavior Scale, Chinese version of Health Behavior Motivation Scale, and Health Literacy Questionnaire) were used with explicit permission obtained via email from the respective copyright holders.

### Investigation methods and quality control

Pre-survey Phase: All investigators possessed nursing or related professional backgrounds. They underwent standardized training on the study instruments, assessment protocols, and precautions to ensure consistent and accurate data collection. Proficiency was confirmed through a qualifying examination.

Survey Phase: A standardized script was used to introduce the questionnaire. Investigators explained scale items using concrete life examples when participants had difficulty understanding, ensuring consistent clarification of any queries. To minimize data loss and ensure validity, all questionnaires were distributed and collected on-site, with immediate review for completeness after participants finished.

Data Entry and Analysis Phase: To ensure accuracy, data were independently entered by two personnel. Any discrepancies were resolved by referring back to the original questionnaires.

### Statistical methods

To ensure data accuracy, a double-entry procedure was performed using EpiData software (version 3.1). All statistical analyses were conducted with IBM SPSS Statistics (version 26.0). A two-sided *P*-value of less than 0.05 was considered statistically significant.

## Results

### General information of the respondents

In this survey, a total of 616 questionnaires were distributed, and 616 valid questionnaires were recovered. The mean age of the respondents was (63.5 ± 10.2) years, and the demographic characteristics of the sample are as follows: the gender distribution is balanced (males 51.8%, females 48.2%); the majority of participants are of Han ethnicity (82.0%), while Yi ethnicity accounts for 15.7%; the age of the sample is concentrated in the range of 50–69 years (totaling 56.5%); 81.7% of respondents have an educational attainment of junior high school or below, 76.1% reside in rural areas, 76.1% are enrolled in the New Rural Cooperative Medical Scheme, 74.2% are complicated with overweight/obesity, 75.3% are complicated with hyperlipidemia, 58.4% never undergo physical examinations, and 38.1% have no knowledge of stroke, Please refer to Table 1.

**Table 1:** Single factor analysis of active health behavior in high risk population of stroke (*n* = 616).

Category	Example (%)	score（$\bar{X}\;\pm \;S$）	*F/t*	*P* value
Gender			−1.65	0.100
Male	319（51.8）	55.40 ± 8.01		
Female	297（48.2）	56.44 ± 7.65		
Ethnicity			1.42	0.242
Han nationality	505（82）	55.92 ± 8.06		
Yi nationality	97（15.7）	55.31 ± 6.65		
Other	14（2.3）	59.07 ± 7.63		
Age			2.65	< 0.032*
40∼49	97（15.7）	57.03 ± 7.68		
50∼59	178（28.9）	54.38 ± 7.88		
60∼69	170（27.6）	56.58 ± 7.49		
70∼79	133（21.6）	56.33 ± 8.24		
≥ 80	38（6.2）	55.55 ± 7.67		
Smoking			−3.64	< 0.000**
Yes	198（32.1）	54.25 ± 7.45		
No	418（67.9）	56.68 ± 7.93		
Drinking alcohol			−1.99	< 0.048*
Yes	222（36）	55.06 ± 7.71		
No	394（64）	56.37 ± 7.90		
Marital status			0.85	0.395
Married	520（84.4）	56.01 ± 7.83		
Unmarried	96（15.6）	55.27 ± 8.00		
Degree of education			0.69	0.502
College or above	42（6.8）	56.31 ± 6.43		
Technical school/High school	71（11.5）	56.85 ± 7.65		
Junior high school or below	503（81.7）	55.73 ± 7.99		
Status of occupation			1.87	0.155
Employed	316（51.3）	55.31 ± 8.06		
Retirement	276（44.8）	56.56 ± 7.66		
Unemployment	24（3.9）	56.04 ± 6.79		
Living arrangements			2.56	0.061
With one's spouse	208（33.8）	57.07 ± 8.14		
With one's children	73（11.9）	55.11 ± 8.67		
With one's spouse and children	282（45.8）	56.11 ± 7.43		
Living alone	53（8.6）	57.11 ± 7.33		
Place of residence			2.56	< 0.011*
City	147（23.9）	57.30 ± 7.51		
Rural areas	469（76.1）	55.46 ± 7.91		
Source of living			7.93	< 0.000**
Retirement pension	195（31.7）	57.73 ± 7.43		
Support for children	109（17.7）	55.10 ± 7.54		
Individual	312（50.6）	55.03 ± 8.04		
Average monthly income			−1.34	0.182
< ¥5,000	598（97.1）	55.82 ± 7.86		
≥ ¥5,000	18（2.9）	58.33 ± 7.39		
Medical costs			1.46	0.145
Employee medical insurance	159（25.8）	56.68 ± 7.19		
New Rural Cooperative Medical Care System	457（74.2）	55.63 ± 8.06		
BMI			0.73	0.535
< 18.5	11（1.8）	53.10 ± 7.69		
18.5∼24.9	202（32.8）	55.95 ± 8.07		
25∼29.9	291（47.2）	55.74 ± 7.39		
≥ 30	112（18.2）	56.50 ± 8.63		
Family history of stroke			0.89	0.372
Yes	193（31.3）	56.32 ± 8.01		
No	423（68.7）	55.71 ± 7.78		
Number of comorbidities			4.06	< 0.003*
No	58（9.4）	54.74 ± 7.52		
1	288（46.8）	55.06 ± 7.81		
2	249（40.4）	57.29 ± 7.79		
3	19（3.1）	53.21 ± 7.55		
≥ 4	2（0.3）	62.50 ± 7.55		
Hyperlipidemia			0.98	0.327
Yes	464（75.3）	56.08 ± 8.09		
No	152（24.7）	55.36 ± 7.08		
High homocysteine			0.09	0.931
Yes	248（40.3）	55.93 ± 8.09		
No	368（59.7）	55.88 ± 7.70		
Frequency of physical examination			13.43	< 0.000**
Several times/year	67（10.9）	61.12 ± 8.41		
Once/year	74（12）	57.09 ± 8.18		
Once every few years	115（18.7）	55.14 ± 6.63		
Never	360（58.4）	54.92 ± 7.65		
Whether to receive training			7.35	< 0.001**
Have been accepted	152（24.7）	57.00 ± 7.91		
Have seen	229（37.2）	56.73 ± 7.93		
Know nothing about	235（38.1）	54.37 ± 7.53		

* indicates *P* value < 0.05, **indicates *P* value < 0.01. BMI stands for body mass index.

### Active health behavior scores in high-risk stroke populations

The total average score of active health behaviors among high-risk groups of stroke was (55.90 ± 7.85) points. The average scores of each dimension item from high to low were as follows: interpersonal support dimension (3.15 ± 0.58), individual health responsibility dimension (2.76 ± 0.72), nutritional dietary behavior dimension (2.78 ± 0.78), psychological stress coping dimension (2.71 ± 0.51), and exercise behavior dimension (2.59 ± 0.79). According to the scale, a score below 60 indicates poor active health behaviors, suggesting that the overall active health behaviors are at a moderately low level. The average score of all items in the active health behavior scale was only (2.79 ± 0.39), However, the average item score for the interpersonal relationship support dimension was (3.15 ± 0.58), which was significantly higher than that of the other dimensions. The possible explanation for this phenomenon is that there might be a bias in the item setting. The items of interpersonal support are mostly perception-based questions, and participants tend to give positive answers. In contrast, dimensions such as exercise and nutrition require actual behaviors, and reports are more conservative, resulting in the score differences among dimensions not directly reflecting the real behavioral differences. Please refer to Table 2.

**Table 2:** Score of active health behavior of stroke high-risk population (*n* = 616, X ± S).

Project	Total score	Average score
Active Health Behavior Scale score	55.90 ± 7.85	2.79 ± 0.39
Individual health responsibility	2.76 ± 0.72	2.76 ± 0.72
Coping with psychological stress	2.71 ± 0.51	2.71 ± 0.51
Interpersonal support	3.15 ± 0.58	3.15 ± 0.58
Exercise behavior	2.59 ± 0.79	2.59 ± 0.79
Nutrition dietary behavior	2.78 ± 0.78	2.78 ± 0.78

### Univariable analysis of active health behaviors in a high-risk stroke population

The results of univariate analysis showed that age had an effect on active health behaviors (*P* < 0.05), with the highest score in the 40∼49 years age group, the lowest score in the 50∼59 years age group, and a slight rebound after 60 years of age. Statistically significant differences in active health behavior scores (*P* < 0.05) were observed across populations stratified by smoking status, alcohol consumption, residential location, source of livelihood, number of chronic comorbidities, frequency of physical examinations, and level of exposure to stroke-related knowledge. Several subgroup analyses involved extremely small sample sizes: only 14 cases (accounting for 2.3%) were from other ethnic minority groups. Although the overall sample sizes of subgroups such as the high homocysteine group and hyperlipidemia group were sufficient, when subdivided by the number of chronic comorbidities, only 2 cases (accounting for 0.3%) had 4 or more chronic comorbidities, only 11 cases (accounting for 1.8%) had low BMI, and only 18 cases (accounting for 2.9%) had an average monthly income of ≥5,000 yuan. The scores of these small-sample groups may be extreme values and are not representative of the population. The absence of significant associations for variables including ethnicity, BMI, and average monthly income in univariate analysis may be attributed to the influence of small sample sizes, which prevents the authentic reflection of the association between these variables and active health behaviors. Please refer to Table 1.

### Correlation analysis of active health behaviors with health behavior motivation, health literacy, social support and self-efficacy among populations at high risk of stroke

The Active Health Behavior Scale for Chronic Diseases shows significant positive correlations with the Health Behavior Motivation Scale (*r* = 0.156, *P* < 0.01), the Medical Social Support Scale (r = 0.412, *P* < 0.01), and the General Self-Efficacy Scale (*r* = 0.401, *P* < 0.01),； On the contrary, the Chronic Disease Active Health Scale is significantly negatively correlated with the Health Literacy Questionnaire (*r* = −0.380, *P* < 0.01). Please refer to Table 3.

**Table 3:** Correlation analysis of active health behaviors among high-risk population for stroke (*n* = 616).

Category	HBMS	HLS-Q16 Chinese version	MOS-SSS	General self-efficacy Scale	PHBS-CD
HBMS	1				
HLS-Q16 Chinese version	−0.139**	1			
MOS-SSS	0.123**	−0.149**	1		
General self-efficacy Scale	0.127**	−0.282**	0.380**	1	
PHBS-CD	0.156**	−0.380**	0.412**	0.401**	1

Pearson correlation analysis: **Correlation is significant at the 0.01 level (2-tailed). *Correlation is significant at the 0.05 level (2-tailed). PHBS-CD, proactive health behavior scale for patients with chronic diseases; HBMS, the Chinese version of health behavior motivation scale; HLS-Q16 Chinese version, Chinese version of the health literacy questionnaire; MOS-SSS, medical outcomes study social support survey.

### Multiple linear regression analysis of factors influencing active health behaviors in a high-risk stroke population

Multiple linear regression analysis was performed with the total score of active health behaviors for chronic diseases as the dependent variable, and variables with statistical significance in univariate analysis as independent variables. Multiple linear regression was applied to explore the influencing factors among populations at high risk of stroke, and the specific variable selection procedure is detailed in Table 4. The results showed that a total of 7 variables, namely health literacy, medical social support, general self-efficacy, age, smoking, source of livelihood, and physical examination frequency, were entered into the regression equation, which could explain 31.8% of the total variance. Among these variables, medical social support and general self-efficacy are positive promoting factors; while age, smoking, higher health literacy score, insecure source of livelihood, and lower physical examination frequency are negative inhibiting factors. Please refer to Table 5.

**Table 4:** Variable allocation table.

Variable	Variable Assignment
Age	The 40–49 years age group was set as the reference (group). 50∼59 (0, 1, 0, 0, 0), 60∼69 (0, 0, 1, 0, 0),70∼79 (0, 0, 0, 1, 0), ≥ 80 (0, 0, 0, 0, 1)
Smoking	Yes = 0, No = 1
Drinking alcohol	Yes = 0, No = 1
Place of residence	City = 0, Rural areas = 1
Source of living	The primary source of living was included as dummy variables, Support for children (0,1,0), Individual (0, 0, 1)
Number of comorbidities	The “No” group was set as the reference (category). 1 (0, 1, 0, 0, 0), 2 (0, 0, 1, 0, 0), 3 (0, 0, 0, 1, 0), ≥ 4 (0, 0, 0, 0, 1)
Frequency of physical examination	“Several times a year” was set as the reference (category). Once/year (0, 1, 0, 0), Once every few years (0, 0, 1, 0), Never (0, 0, 0, 1)
Whether to receive training	Those who had received stroke-related health education were set as the reference category. Have seen (0, 1, 0), Know nothing about (0, 0, 1)
HBMS Chinese version	Original value entry
HLS-Q16 Chinese version	Original value entry
MOS-SSS	Original value entry
General self-efficacy Scale	Original value entry

HBMS, the Chinese version of health behavior motivation scale; HLS-Q16 Chinese version, Chinese version of the health literacy questionnaire; MOS-SSS, medical outcomes study social support survey.

**Table 5:** Multiple linear regression analysis of influencing factors of active health behavior in high-risk population of stroke (*n* = 616).

Category	*B* value	*S Evalue*	*β*	*t*	*P*	tolerance	VIF
HBMS Chinese version	0.062	0.032	0.064	1.944	0.052	0.924	1.082
HLS-Q16 Chinese version	−0.599	0.088	−0.231	−6.798	< 0.000	0.872	1.147
MOS-SSS	0.313	0.038	0.289	8.176	< 0.000	0.804	1.244
General self-efficacy Scale	0.446	0.08	0.203	5.546	< 0.000	0.754	1.326
Age							
50∼59	−2.06	0.791	−0.119	−2.605	< 0.009	0.482	2.074
Smoking							
No	1.471	0.606	0.088	2.426	< 0.016	0.773	1.293
Source of living							
Support for children	−2.031	0.786	−0.099	−2.583	< 0.010	0.688	1.453
Individual	−1.777	0.682	−0.113	−2.606	< 0.009	0.533	1.877
Frequency of physical examination							
Once/year	−1.901	1.078	−0.079	−1.763	0.078	0.504	1.983
Once every few years	−2.384	1.014	−0.118	−2.35	< 0.019	0.397	2.521
Never	−3.457	0.874	−0.217	−3.954	< 0.000	0.334	2.996

R^2^ = 40.3%, adjusted R^2^ = 31.8%, F = 18.206, *P* < 0.001; HBMS, the Chinese version of health behavior motivation scale; HLS-Q16 Chinese version, Chinese version of the health literacy questionnaire; MOS-SSS, medical outcomes study social support survey.

## Discussion

### The level of active health behaviors in the high-risk stroke population was relatively low

With corresponding changes in lifestyles and dietary structures, the onset age of stroke has shown a trend toward younger ages. The findings of this study indicate that the average score of active health behaviors among the high-risk stroke population was (55.90 ± 7.85). According to the reference criteria of the scale, an overall score lower than 60 indicates a poor level of active health behaviors, suggesting that the active health behavior level of this population needs further improvement. In the sample of this study, rural populations accounted for 76.1%, individuals with junior high school education or below accounted for 81.7%, and unemployed/retired individuals accounted for 48.7%, meaning the sample is overall composed of groups with low socioeconomic status, which may lead to limitations in sample representativeness. Consistent with the findings of previous studies by Lamelas (21) and Hamid (22), this population often has limited access to health information and relatively weak health awareness and may neglect health behaviors due to economic pressure. Future studies can specifically conduct research on active health behaviors among high-risk stroke populations in urban areas and populations with higher educational levels to avoid variable effect bias caused by single-population research, clarify the behavioral driving factors of populations with different socioeconomic statuses, and provide more comprehensive data support for stroke prevention policies.

### Active health behaviors in high-risk stroke populations are influenced by multidimensional factors

This study confirms that active health behaviors in high-risk stroke populations are not determined by a single factor but result from interactions between individual intrinsic factors and external environmental factors.

### Targeted lifestyle interventions for high-risk stroke populations across different age groups

In this study, individuals aged 40∼49 exhibited the highest scores for proactive health behaviors. However, this finding diverges from the earlier conclusion by Lei (23), which reported elevated health behavior levels among elderly females. The observed phenomenon suggests that individuals in the 40∼49 age bracket are at a critical transitional phase where physiological functions begin to decline. The initial detection of subclinical risks, such as hypertension, hyperglycemia, and hyperlipidemia during routine health examinations elicits a heightened perception of health threats, thereby activating primary prevention motivation grounded in fear appeals. Additionally, this study identified that smokers, as a high-risk group, demonstrated lower proactive health behavior scores, whereas Zhu Tianshun (24) reported no association between smoking and proactive health behaviors. A plausible explanation for this discrepancy may lie in the high-stress context of midlife, where the adoption of health-related practices is constrained by limited time and energy.

### Differential effects of self-efficacy and health literacy on active health behaviors and the mediating moderation mechanism

Correlation analysis in this study demonstrates that scores on the General Self-Efficacy Scale are positively correlated with both the Medical Social Support Scale (*r* = 0.380, *P* < 0.01) and the Health Behavior Motivation Scale (*r* = 0.127, *P* < 0.01). Moreover, self-efficacy exerts a significant effect on behavior (β = 0.203, *P* < 0.001), which is consistent with the findings reported by Chen (25). This indicates that social support can improve self-efficacy and that social support constitutes a mediating pathway for promoting active health behaviors. Meanwhile, self-efficacy may also moderate the relationship between health literacy and behavior: individuals with high self-efficacy are more willing to adopt health behaviors even when their health literacy is at a relatively low level, thereby attenuating the negative impact of low health literacy. The present study identifies a negative effect of health literacy on active health behaviors, which contradicts the positive effect reported in the study by Zeng (26). This finding contradicts conventional wisdom. A plausible explanation for this discrepancy is that the proportion of individuals with high health literacy is relatively low in the present study, and the attention that these participants pay to knowledge regarding healthcare, disease prevention, and health promotion only remains at the cognitive level, without being translated into actual behavior (27).

### Translating health literacy into practice and enhancing self-efficacy to improve active health behaviors

This study revealed that high-risk populations supported by their children obtained the lowest scores for active health behaviors, with 58.4% of individuals in this group never undergoing physical examinations. A potential explanation for this observation is twofold: on one hand, low income constrains households from making forward-looking investments in medical and health domains, making it difficult to access high-quality medical resources and reliable channels for health information (28); on the other hand, the uneven distribution of rural medical resources, coupled with high transportation and time costs, hinders the acquisition of self-management knowledge and skills (29). The findings of this study are consistent with those reported by Yang (30), which indicated that unemployed groups suffer from certain economic, livelihood and psychological pressures, leading to insufficient attention to their own health or inadequate capacity to afford health-related consumption. Therefore, medical practitioners should analyze the joint impacts of multiple factors including medical insurance reimbursement ratios, distribution of primary medical resources, and income levels on the utilization rate of stroke prevention services, so as to promote the improvement of medical policies.

### Translate knowledge acquisition into motivated behavior and enhance the willingness for proactive health actions

In this study, 38.1% of participants had no knowledge of stroke; only 24.7% had received formal education on stroke-related knowledge, leading to an extremely low coverage rate of information dissemination. Univariate analysis showed that the proactive health behavior score of the “no knowledge of stroke” group (54.37 ± 7.53) was lower than that of the group who had received stroke-related education (57.00 ± 7.91). However, the variable “whether having received formal training or self-studied stroke knowledge” was excluded from multiple regression analysis. A possible explanation for the findings of this study is that the form of information dissemination is unitary, and the group that has only “seen others suffer from stroke” has not formed systematic cognition of stroke. This result is consistent with the previous study by Noguchi (31), which indicated that rural populations and populations with low educational attainment have difficulty understanding professional medical knowledge; thus, knowledge cannot be converted into behavioral motivation. Therefore, medical personnel should timely evaluate the disease cognition of this population and motivate and activate their intrinsic self-management motivation so as to improve their proactive health behaviors.

### Medical social support exerts a positive effect on active health behaviors and has predictive capacity

The findings of this study demonstrate that the total score of the Medical Social Support Scale is significantly positively correlated with active health behaviors, with a standardized regression coefficient of（β=0.203, *P* < 0.001), indicating that medical social support is a positive contributing factor to active health behaviors. Meanwhile, correlation analysis confirms that the score of the Active Health Behavior Scale is significantly positively correlated with the score of the Medical Social Support Scale (*r* = 0.412, *P* < 0.01). Furthermore, an analysis of scores across each dimension of active health behaviors shows that the mean item score for the interpersonal support dimension is (3.15 ± 0.58), which is higher than the scores of dimensions including personal health responsibility, psychological stress coping, exercise behavior and dietary behavior. These findings suggest that, on one hand, rural groups with low educational attainment generally have relatively homogeneous social networks and thus rely more heavily on family support and community medical resources (32); on the other hand, emotional care promotes active health behaviors more effectively than pure material assistance (33), although this result may also be affected by perception bias induced by item design. This is consistent with the findings reported by Wan (34), Cheng (35) and other scholars, which further verifies that in health promotion behavior intervention, support strategies centered on emotional support outperform the traditional model that only focuses on resource provision. Considering the characteristic of the study sample that rural populations and groups with low educational attainment account for a relatively large proportion, future research can explore the differences in the effects of three sources of support, namely family support, community support and medical support, on active health behaviors respectively.

### Limitations

This study has several limitations that should be acknowledged. First, although the study included ten townships, all participants were from the Panzhihua area in Sichuan Province. This geographical restriction may limit the generalizability of the findings to other regions of Sichuan Province. Second, the cross-sectional design of the study impedes the ability to establish causal inferences or examine longitudinal changes in active health behaviors. Third, the measurement of active health behaviors in high-risk stroke populations lacks a well-validated specific scale; this study adopted a scale originally designed for chronic disease populations, which may not fully capture the conceptual nuances of the target construct. Additionally, the sample was predominantly composed of rural residents (76.1%) and low-income individuals (97.1% with monthly incomes below ¥5,000), which may limit the applicability of the results to more economically developed or urbanized settings. Future research should adopt a multi-center longitudinal design with follow-up assessments to enhance the external validity of the findings.

## Conclusion

This study demonstrates that the overall level of active health behaviors among people at high risk of stroke in Panzhihua remains at a moderately low level. The influencing factors include both non-modifiable factors such as age, and modifiable factors covering smoking, alcohol consumption, source of livelihood, frequency of physical examinations, health literacy, medical social support, self-efficacy and other variables. Comprehensive and individualized intervention strategies should be implemented in the future: first, health education should be adopted to improve health literacy and self-efficacy, and strengthen intrinsic behavioral motivation; second, a three-tier support network of “family-community-medical institution” should be constructed to optimize the external support environment, with a focus on promoting the management and control of underlying diseases such as hypertension and dyslipidemia, popularizing healthy lifestyles, effectively improving the active health behaviors of high-risk groups, and reducing the risk of stroke incidence. This study may have limitations including small sample bias; nevertheless, the identified status quo and underlying mechanisms still provide critical scientific evidence for subsequent research on active health behaviors and the development of intervention programs for this population.

## Data Availability

The original contributions presented in the study are included in the article/Supplementary Material, further inquiries can be directed to the corresponding author.
